# Lactate dehydrogenase predicts disease progression outcome in COVID-19 patients treated with Azvudine

**DOI:** 10.3389/fcimb.2023.1237277

**Published:** 2023-10-18

**Authors:** Manyun Mao, Yating Dian, Yuming Sun, Wangqing Chen, Wu Zhu, Guangtong Deng

**Affiliations:** ^1^ Department of Dermatology, Xiangya Hospital, Central South University, Changsha, China; ^2^ National Engineering Research Center of Personalized Diagnostic and Therapeutic Technology, Changsha, Hunan, China; ^3^ Furong Laboratory, Changsha, Hunan, China; ^4^ Hunan Key Laboratory of Skin Cancer and Psoriasis, Hunan Engineering Research Center of Skin Health and Disease, Xiangya Hospital, Central South University, Changsha, China; ^5^ National Clinical Research Center for Geriatric Disorders, Xiangya Hospital, Central South University, Changsha, Hunan, China; ^6^ Department of Plastic and Cosmetic Surgery, Xiangya Hospital, Central South University, Changsha, China

**Keywords:** azvudine, lactate dehydrogenase, COVID-19, prognosis, SARS-CoV-2

## Abstract

**Background:**

Azvudine has been approved in China for the treatment of COVID-19 patients. Previous studies have suggested a correlation between high levels of lactate dehydrogenase (LDH) and the severity of COVID-19. However, the impact of LDH levels in COVID-19 patients receiving Azvudine treatment remains unclear.

**Methods:**

In this retrospective cohort study, we analyzed the data of 351 hospitalized COVID-19 patients who were consecutively treated with Azvudine, with or without high LDH levels. The clinical features, treatment strategies and prognosis data were collected and analyzed.

**Results:**

Among the 351 hospitalized patients with COVID-19 treated with Azvudine (119 with high-LDH levels), the median age was 69 years (range 58–78), and 213 (60.7%) were male. Common symptoms included cough (86.0%), expectoration (73.5%), fever (69.8%), polypnea (47.6%) and poor appetite (46.4%). Patients with high LDH levels exhibited significantly elevated leucocyte and neutrophil counts, elevated level of myocardial enzymes, as well as higher levels of inflammatory markers such as interleukin-6, interleukin-10, procalcitonin, C reactive protein, ferritin, and prolonged erythrocyte sedimentation rate upon admission. COVID-19 patients with high-LDH levels had higher rates of corticosteroid therapy, non-invasive and invasive mechanical ventilation, worsened and death (2.5% vs. 0%). The Cox proportional hazard model demonstrated that high LDH levels (adjusted hazard ratio = 5.27; 95% confidence interval: 1.19, 14.50) were associated with a more unfavorable composite disease progression outcome among COVID-19 patients treated with Azvudine, after accounting for potential confounding variables.

**Conclusion:**

High-LDH levels predict a worse composite disease progression outcome in COVID-19 patients treated with Azvudine.

## Introduction

Coronavirus disease 2019 (COVID-19), caused by the infection of Severe Acute Respiratory Syndrome Coronavirus 2 (SARS-CoV-2), has emerged as a significant global public health threat in recent years (The, 2020; The, 2023). Azvudine, a nucleoside analog that inhibits HIV-1 RNA-dependent RNA polymerase (Yu and Chang, 2020), has shown promise in combating COVID-19. Zhang et al. discovered during the 2021 COVID-19 outbreak that oral administration of Azvudine effectively inhibits SARS-CoV-2 replication, preserves thymus immune function, and provides rapid treatment for COVID-19 patients (Zhang et al., 2021). In July 2022, the China National Medical Products Administration and the National Health Commission of China approved Azvudine for the treatment of adult patients with mild COVID-19 (Yu and Chang, 2022). Several subsequent clinical trials, including our own previous study, demonstrated the effectiveness of oral Azvudine in curing COVID-19 patients (Ren et al., 2020; Zhang et al., 2021; Yu and Chang, 2022; Deng et al., 2023; Shen et al., 2023; Sun et al., 2023). However, the association between inflammatory biomarkers and prognosis in COVID-19 patients undergoing Azvudine treatment remains unclear.Lactate dehydrogenase (LDH), a cytoplasmic glycolytic enzyme found in almost every tissue, is commonly used as an indicator of tissue damage (Jurisic et al., 2015). Numerous studies have shown that elevated LDH levels are positively associated with the severity of COVID-19 (Bartziokas and Kostikas, 2021; Szarpak et al., 2021; Alonso-Bernáldez et al., 2023; Ergenc et al., 2023). However, it is still uncertain whether LDH can predict the prognosis in COVID-19 patients receiving Azvudine treatment. In the present retrospective study, we reviewed the clinical data of 351 adult patients with positive RT-PCR for SARS-CoV-2 infection who were treated with Azvudine. We compared the clinical characteristics, laboratory markers and short-term prognosis, including mortality, between patients with high-LDH levels and those without. The aim of this study was to investigate the role of high-LDH levels as a predictive marker of response to Azvudine treatment in COVID-19 patients.

## Methods

### Study design

We conducted a single-center, retrospective cohort study of hospitalized adult patients with positive RT-PCR for SARS-CoV-2 infection, who were given Azvudine in Xiangya Hospital, during the period from December 5, 2022 to January 26, 2023. We excluded patients who were younger than 18 years; those without LDH test results; those have a low LDH (<50U/L) or those who did not receive Azvudine treatment. This study was approved by the institutional review board of Xiangya Hospital, Central South University (202002024), and individual patient-informed consent was not required for this retrospective cohort study using anonymized data.

### Data source

The electronic health records of COVID-19 patients were retrieved from the inpatient system of Xiangya Hospital. These comprehensive records include various details such as demographic information, admission records, diagnoses, prescribed medications, drug dispensing records, procedures, laboratory tests, and dates of discharge or death. The health records were then linked with anonymized vaccination records provided by the Department of Immunization, Center for Disease Control and Prevention of Hunan Province using unique identification numbers (China Identity Card).

### Definition of conditions

LDH levels were assessed using the lactate dehydrogenase substrate method (Beckman AU5800). The values of LDH were collected as continuous variables and analyzed in binary form. Patients were categorized into two groups based on their LDH levels. The normal-LDH group included patients with LDH values ranging from 50 to 250 U/L, while the high-LDH group included patients with LDH values greater than 250 U/L. Severe COVID-19 patients were defined as having respiratory rate ≥30 times per minute, or oxygen saturation ≤ 93%, or PaO2/FiO2 ≤300 mmHg, or lung infiltrates >50% on admission.

### Statistical analysis

Descriptive statistics were conducted to summarize all variables in the study. Categorical variables were compared using the Fisher exact test or χ2 test, while continuous variables were compared using the t test or the Mann-Whitney U test, as appropriate, to evaluate the study outcome. Continuous variables were presented as mean (SD) or median [interquartile range (IQR)] values, while categorical variables were presented as proportions. Disease progression outcome was depicted using the Kaplan-Meier method and compared between patients with normal LDH levels or with high LDH levels using the log-rank test. Multivariate Cox regression models were used to determine the independent risk factors for disease progression during hospitalization. The adjusted hazard ratio (aHR) was calculated to assess the risk factors. Statistical analysis was conducted using SPSS version 25.0 (IBM) and R version 4.2.1., and statistical charts were generated using Excel 2016 (Microsoft). The significance level was set at P < 0.05 for all statistical analyses.

## Results

### Baseline characteristics of COVID-19 patients

**Figure 1:**
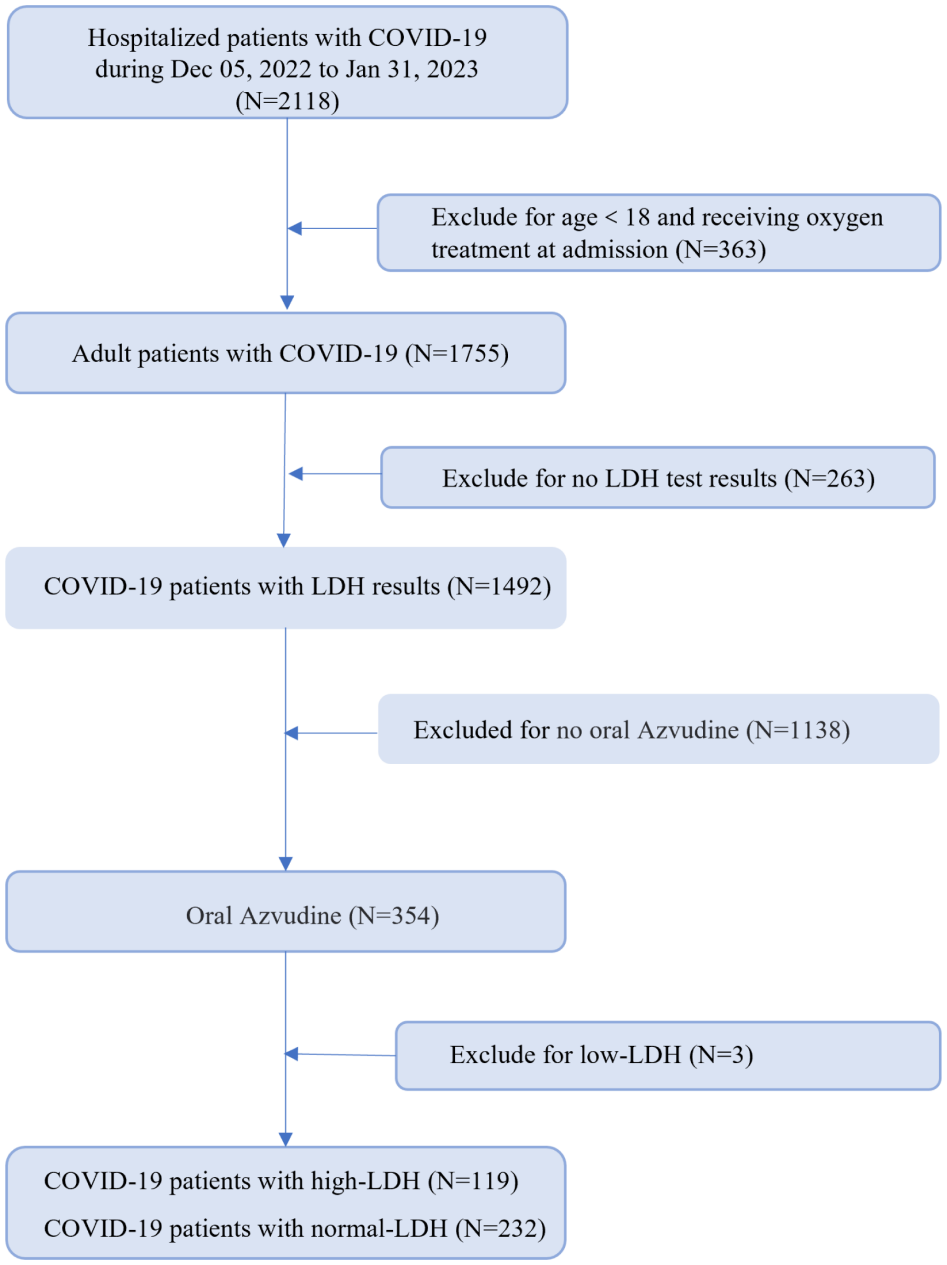
The flow chart of this study.

We collected and analyzed information from 351 hospitalized patients with COVID-19. All patients underwent LDH testing, received Azvudine treatment, and were followed up for a period of 30 days. More than half of the patients (51.3%) had high LDH levels (**Figure 1**). The demographic and clinical characteristics of the patients on admission were summarized in **Table 1**. The median age was 69 years (IQR, 58–78), and 213 (60.7%) were male. Common symptoms at the onset of illness were dry cough (302, 86.0%), expectoration (258, 73.5%), fever (245, 69.8%), polypnea (167, 47.6%) and poor appetite (163, 46.4%). Comorbidities were present in more than half of the patients. Cardiovascular diseases were the most common comorbidity, with 198 patients (69.5%) having this condition. Other comorbidities included endocrine system disease (89, 31.2%), nervous system disease (42, 14.7%), infectious diseases (29, 10.0%), urinary system diseases (33, 11.6%), cancer (28, 8.0%), post-operative conditions (24, 8.4%), and immune system disease (8, 2.0%). There were no significant differences in gender and age between the normal-LDH and high-LDH groups of patients. Regarding COVID-19-related clinical symptoms, patients with high-LDH were more likely to experience poor appetite (54.6% vs. 42.2%, P = 0.028) and palpitation (5.2% vs. 0.0%, P = 0.010) compared to those with normal-LDH levels. However, there were no significant differences in the proportions of other clinical symptoms between the two groups. In terms of preexisting conditions, the high-LDH group had a higher prevalence of hypertension compared to the normal-LDH group (54.6% vs. 43.5%, P = 0.049). However, there were no significant differences in the prevalence of other diseases such as coronary disease, Percutaneous Coronary Intervention, or post-pacemaker surgery between the two groups.

**Table 1:** Characteristics of the patients with COVID-19 treated with Azvudine.

Characteristics	Total (N=351)	Normal-LDH (n=232)	High -LDH (n=119)	*P*
Age (year), median (IQR)	69(58, 78)	68(56.25, 77)	70(59, 79)	0.073
Sex, n (%)				0.680
Men	213(60.7)	139(59.9)	74(62.2)	
Women	138(39.3)	93(40.1)	45(37.8)	
Symptoms, n (%)				
Fever	245(69.8)	163(70.3)	82(68.9)	0.794
Dry cough	302(86.0)	200(86.2)	102(85.7)	0.900
Expectoration	258(73.5)	167(72.0)	91(76.5)	0.367
Poor appetite	163(46.4)	98(42.2)	65(54.6)	**0.028**
Polypnea	167(47.6)	105(45.3)	62(52.1)	0.224
Fatigue	106(30.2)	75(32.3)	31(26.1)	0.225
Stuffiness	97(27.6)	62(26.7)	35(29.4)	0.594
Myalgia	64(18.2)	42(18.1)	22(18.5)	0.930
Headache	35(10.0)	26(11.2)	9(7.6)	0.281
Dyspnea	30(8.5)	18(7.8)	12(10.1)	0.451
Celialgia	20(5.7)	10(4.3)	10(8.4)	0.117
Pharyngalgia	27(7.7)	20(8.6)	7(5.9)	0.362
Dizzy	15(4.3)	10(4.3)	5(4.2)	0.962
Vomiting	15(4.3)	7(3.0)	8(6.7)	0.104
Chest pain	13(3.7)	8(3.4)	5(4.2)	0.769
Nausea	12(3.4)	6(2.5)	6(5.0)	0.232
Palpitation	12(3.4)	12(5.2)	0(0)	**0.010**
Disturbance of consciousness	5(1.4)	3(1.3)	2(1.7)	1.000
Hyposmia	5(1.4)	4(1.7)	1(0.8)	0.666
Hypogeusia	3(0.9)	2(0.9)	1(0.8)	1.000
Diarrhea	2(0.6)	2(0.9)	0(0)	0.551
Joint sore	2(0.6)	1(0.8)	1(0.4)	1.000
Preexisting condition, n (%)				
Cardiovascular diseases	198(69.5)	119(66.1)	79(75.2)	0.107
Hypertension	166(47.3)	101(43.5)	65(54.6)	**0.049**
Coronary disease	85(24.2)	51(22.0)	34(28.6)	0.173
PCI	14(7.1)	7(5.9)	7(8.9)	0.423
Post-pacemaker surgery	2(1.0)	1(0.8)	1(1.3)	1.000
Endocrine system disease	89(31.2)	57(31.7)	32(30.5)	0.834
DM	81(23.1)	50(21.6)	31(26.1)	0.344
Hyperthyreosis	7(7.9)	6(10.5)	1(3.1)	0.414
Hypothyroidism	1(1.1)	1(1.8)	0(0)	1.000
Chronic respiratory disease	40(14.0)	29(16.1)	11(10.5)	0.186
COPD	21(6.0)	15(6.5)	6(5.0)	0.595
Asthma	5(12.5)	4(13.8)	1(9.1)	1.000
Nervous system disease	42(14.7)	23(12.8)	19(18.1)	0.222
Infectious diseases	29(10.2)	19(10.6)	10(9.5)	0.781
Urinary system diseases	33(11.6)	18(10.0)	15(14.3)	0.275
Cancer	28(8.0)	16(6.9)	12(10.1)	0.297
Post-operative	24(8.4)	17(9.4)	7(6.7)	0.415
Immune system disease	8(2.8)	5(2.8)	3(2.9)	1.000

LDH, lactic dehydrogenase; IQR, interquartile range; DM, diabetes mellitus; COPD, chronic obstructive pulmonary disease.

The bold values/numbers are intended to highlight results with statistical significance (P≤0.05, P≤0.01 or P≤0.001).

### Laboratory findings on admission

The laboratory findings on admission of the COVID-19 patients with or without high-LDH levels were presented in **Table 2**. The majority of patients (76.3%) had a normal leukocyte count, while 13.1% had an increased leukocyte count and 10.4% had a decreased leukocyte count. Patients with high LDH levels had a higher median neutrophil count and neutrophil percentage, but a lower lymphocyte count, lymphocyte percentage, eosinophil count, and basophil count. Remarkably, patients with high LDH levels also showed significantly higher serum concentrations of interleukin-6 (IL-6), interleukin-10 (IL-10), procalcitonin (PCT), C-reactive protein (CRP), and ferritin. Additionally, they exhibited a prolonged erythrocyte sedimentation rate (ESR), indicating a more pronounced inflammatory response in individuals with high LDH levels. These compelling findings provide valuable insights into the association between LDH and markers of inflammation, suggesting that LDH may serve as an indicator of heightened systemic inflammation in patients with COVID-19. Additionally, patients with elevated LDH levels exhibited higher concentrations of organ function markers, including direct bilirubin (DBIL), alanine aminotransferase (ALT), aspartate aminotransferase (AST), serum creatinine (SCr), blood urea nitrogen (BUN), creatine kinase (CK), creatine kinase MB isoenzyme (CK-MB), myoglobin, troponin, and B-type natriuretic peptide (BNP), compared to patients with normal LDH levels. Regarding coagulation function markers, patients with high LDH levels demonstrated increased levels of D-dimer and fibrinogen (FIB) compared to those with normal LDH levels.

**Table 2:** Laboratory findings of COVID-19 patients treated with Azvudine at admission.

Variable	Total (N=351)	Normal-LDH (n=232)	High -LDH (n=119)	*P*
Blood routine index				
WBC (10^9^/L), median (IQR)	5.5(4.3, 7.525)	5.5(4.325, 7)	5.8(4.3, 8.375)	0.169
>9.5×10^9^/L, n (%)	46(13.3)	24(10.5)	22(18.8)	**0.049**
3.5–9.5×10^9^/L, n (%)	264(76.3)	181(79.4)	83(70.3)	
<3.5×10^9^/L, n (%)	36(10.4)	24(10.5)	12(10.2)	
Blood platelet (10^9^/L), median (IQR)	193.5(136.75, 246.5)	195.5(147, 259.75)	187.5(121.75, 224.5)	**0.016**
Neutrophil (10^9^/L), median (IQR)	3.9(2.8, 5.725)	3.7(2.8, 5.075)	4.35(3.0, 7.0)	**0.005**
Neutrophil percentage (%), median (IQR)	72.8(62.7, 81.05)	69.7(60.625, 78.25)	78.1(68.5, 83.625)	**<0.001**
Lymphocyte (10^9^/L), median (IQR)	0.9(0.6, 1.3)	1.0(0.7, 1.3)	0.8(0.5, 1.1)	**<0.001**
<1.1×10^9^/L, n (%)	228(65.9)	137(60.1)	91(77.1)	**0.002**
Lymphocyte percentage (%), median (IQR)	16.55(10, 24.525)	18.4(11.975, 25.9)	12.55(8.15, 19.45)	**<0.001**
<20%, n (%)	222(64.2)	131(57.5)	91(77.1)	**<0.001**
Eosinophil (10^9^/L), median (IQR)	0.02(0, 0.09)	0.4(0.01, 0.10)	0(0, 0.04)	**<0.001**
<0.02×10^9^/L, n (%)	181(52.3)	97(42.5)	84(71.2)	**<0.001**
Basophil (10^9^/L), median (IQR)	0.01(0.01, 0.02)	0.1(0.01, 0.03)	0.1(0, 0.02)	**<0.001**
Hepatorenal function+E4A, median (IQR)				
TBil (μmol/L)	10.1(7.7, 13.05)	10.4(7.4, 13.5)	10.9(8.1, 16.6)	0.099
DBIL (μmol/L)	3.6(2.8, 4.7)	3.8(2.8, 5.0)	4.2(2.8, 6.0)	**0.031**
Albumin (g/L)	34.6(31.8, 37.25)	34.5(31.6, 37.2)	32.8(29.7, 35.9)	**<0.001**
Globulin (g/L)	27.9(25.35, 31.15)	28.1(25.6, 31.5)	30.5(27.1, 32.5)	**0.011**
ALT (U/L)	24.1(15.9, 39.75)	21.3(13.9, 29.3)	25.9(18.1, 55.8)	**0.002**
AST (U/L)	27.4(19.8, 39.225)	24.6(17.9, 31.7)	35.2(28.2, 56.0)	**<0.001**
SCr (μmol/L)	69.65(59, 85.35)	68.0(58.2, 85.3)	74.3(65.3, 106.4)	**0.005**
BUN (mmol/L)	5.605(4.165, 7.578)	5.81(4.14, 7.51)	6.61(5.05, 12.16)	**0.005**
K^+^ (mmol/L)	3.93(3.66, 4.22)	3.84(3.65, 4.15)	3.77(3.53, 4.12)	0.709
Ca^+^(mmol/L)	2.12(2.04, 2.19)	2.12(2.04, 2.19)	2.06(1.98, 3.14)	<0.001
Na^+^(mmol/L)	139.7(137.3, 141.9)	140.1(137.8, 142.6)	139.0(136.6, 142.1)	**0.044**
Cl^-^(mmol/L)	103.7(101,105.8)	104.0(101.8, 106.0)	102.7(99.2, 104.6)	0.118
Mg^+^(mmol/L)	0.86(0.79, 0.91)	0.86(0.78, 0.91)	0.87(0.82, 0.93)	0.141
Myocardial enzyme, median (IQR)				
LDH (U/L)	218.9(183.7, 272.0)	196(173.4, 217.0)	313(273.5, 370.5)	**<0.001**
CK (U/L)	59.5(37.7, 102.2)	52.1(35.1, 78.6)	112.4(68.0, 182.7)	**<0.001**
CK-MB (U/L)	10.3(7.7, 13.3)	9.7(7.0, 12.7)	12.1(9.6, 16.5)	**<0.001**
Myoglobin (U/L)	59.8(40.5, 100.0)	56.1(39.5, 81.8)	128.5(79.0, 223.2)	**<0.001**
Troponin (U/L)	0.005(0.002, 0.013)	0.004(0.002, 0.015)	0.015(0.006, 0.023)	**<0.001**
BNP (ng/ml)	263.07(125.45, 703.89)	223.7(122.9, 536.8)	572.3(212.0, 1920.6)	**<0.001**
Coagulation function, median (IQR)				
D-Dimer (mg/mL)	0.18(0.09, 0.37)	0.14(0.08, 0.28)	0.26(0.14, 0.50)	**<0.001**
PT (sec)	11.5(11.0, 12.5)	11.5(11.0, 12.4)	11.5(11.0, 12.6)	0.407
INR	0.99(0.94, 1.05)	0.98(0.94, 1.05)	0.99(0.94, 1.08)	0.325
FIB (mmol/L)	4.395(3.47, 5.49)	4.02(3.29, 5.16)	4.84(4.16, 5.86)	**<0.001**
APTT (sec)	27.8(25.1, 30.4)	27.5(25.1, 29.7)	28.3(25.0, 31.8)	0.054
TT (sec)	15.8(15.1, 17.0)	15.8(15.1, 17.0)	15.6(15.1, 17.1)	0.857
Inflammatory factor, median (IQR)				
TNF-α (pg/ml)	9.21(4.68, 12.90)	8.87(5.30, 12.40)	9.76(3.79, 14.35)	0.340
IL-2 (pg/ml)	1.995(1.33, 3.145)	2.11(1.58, 3.30)	1.71(1.12, 2.70)	0.241
IL-6 (pg/ml)	9.38(2.81, 22.20)	6.50(2.45, 14.90)	16.78(5.25, 39.33)	**<0.001**
IL-10 (pg/ml)	5.0(3.91, 5.27)	5.0(3.6, 5.0)	5.1(4.6, 9.50)	**0.003**
Other biochemical indexes, median (IQR)				
HBA1c (%)	6.3(5.9, 6.9)	6.4(5.8, 7.0)	6.2(6.1, 6.9)	0.878
Lactic acid (mmol/L)	1.41(1.07, 2.03)	1.31(1.02, 2.13)	1.45(1.27, 1.84)	0.404
PCT (ng/ml)	0.05(0.05, 0.09)	0.05(0.05, 0.06)	0.73(0.05, 0.17)	**<0.001**
CRP (mg/L)	23.7(7.7, 75.9)	14.8(4.7, 51.9)	61.7(20.8, 93.3)	**<0.001**
ESR (mm/h)	54.0(36.5, 71.5)	50.0(32.0, 67.0)	63.0(41.0, 83.0)	**0.015**
Ferritin (ng/ml)	853.3(458.3, 1257)	657.0(420.2, 1062.0)	1042.0(681.9, 1666.8)	**0.007**

DM, diabetes mellitus; IQR, interquartile range; WBC, white blood cell; TBil, total bilirubin; DBIL, direct bilirubin; ALT, alanine aminotransferase; SCr, serum creatinine; BUN, blood urea nitrogen; LDH, lactate dehydrogenase; CK, creatine kinase; CK-MB, creatine kinase isoenzyme MB; BNP, Brain natriuretic peptide precursor; PT, Prohemase time; INR, international normalized ratio; FIB, fibrinogen; APTT, activated partial thromboplastin time; TT, thrombin time; HBA1c, glycated hemoglobin; PCT, procalcitonin; CRP, C-reactive protein; ESR, erythrocyte sedimentation rate.

The bold values/numbers are intended to highlight results with statistical significance (P≤0.05, P≤0.01 or P≤0.001).

### Analysis of severity, treatment and prognosis of patients with COVID-19

We compared the severity, treatment, and short-term prognosis between COVID-19 patients with normal lactate dehydrogenase (LDH) levels and those with high LDH levels, as shown in **Table 3**. While no significant difference in severity was observed between the two groups, it was evident that patients with high LDH levels had a higher utilization of corticosteroid therapy (65.5% vs. 48.7%, P = 0.003), mask oxygen support (8.4% vs. 1.3%, P = 0.002), and high-flow oxygen support (8.4% vs. 1.7%, P = 0.007). Furthermore, patients with high LDH levels exhibited a higher rate of deterioration and fatality (1.7% vs. 0.9%, 2.5% vs. 0, P = 0.041) compared to those with normal LDH levels.

**Table 3:** Disease severity, treatment, and prognosis of COVID-19 patients treated with Azvudine.

Variable	Total (N=351)	Normal-LDH (n=232)	High -LDH (n=119)	*P*
Severity, n (%)				
Mild to moderate	135(38.5)	86(37.1)	49(41.2)	0.454
Severe	216(61.5)	146(62.9)	70(58.8)	
Medication, n (%)				
Immunoregulator	93(26.5)	55(23.7)	38(31.9)	0.098
Corticosteroid	191(54.4)	113(48.7)	78(65.5)	**0.003**
Antibiotics	266(75.8)	171(73.7)	95(78.9)	0.205
Oxygen support, n (%)				
Nasal cannula	316(90.0)	208(89.7)	108(90.8)	0.744
Mask oxygen	13(3.7)	3(1.3)	10(8.4)	**0.002**
High-flow oxygen	14(4.0)	4(1.7)	10(8.4)	**0.007**
Invasive mechanical ventilation	4(1.1)	1(0.4)	3(2.5)	0.115
Prognosis, n (%)				
Discharged	344(98.0)	230(98.1)	114(95.8)	**0.041**
Worsened	4(1.1)	2(0.9)	2(1.7)	
Death	3(0.9)	0(0)	3(2.5)	

The bold values/numbers are intended to highlight results with statistical significance (P≤0.05, P≤0.01 or P≤0.001).

### Analysis of the association between high-LDH and composite disease progression outcome in COVID-19 patients

**Figure 2:**
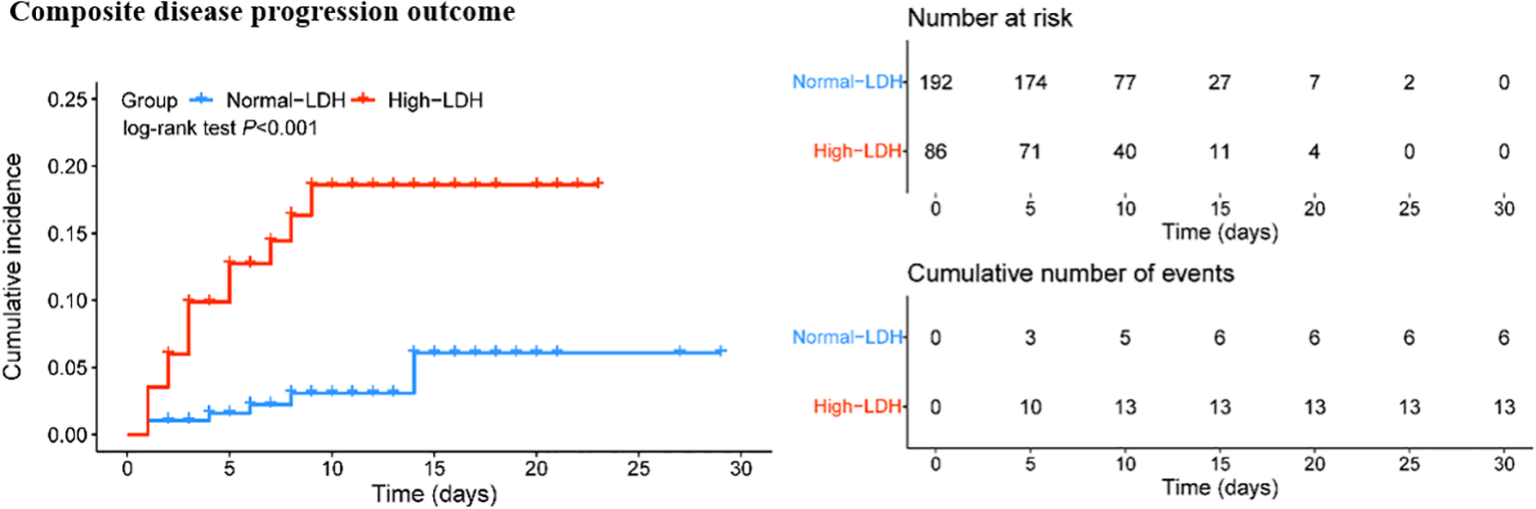
Cumulative incidence of composite disease progression outcome for high LDH levels and normal LDH levels.

The Kaplan-Meier survival curve and Cox proportional hazard model were used to further assess the association between high LDH levels and the composite disease progression outcome of COVID-19. The results showed that COVID-19 patients with high LDH levels had a significantly higher cumulative incidence of exacerbation of disease progression than those with normal LDH levels (P < 0.001) (**Figure 2**). More importantly, high LDH levels were found to be an independent risk factor for the composite disease progression outcome in COVID-19 patients treated with Azvudine, after adjusting for age and sex only (aHR = 4.68; 95% CI: 1.78, 12.34; P = 0.002), or adjusting for steroid and antibiotics (aHR = 4.98; 95% CI: 1.88, 13.16; P = 0.001), or additionally adjusting for age, sex, severity, time from symptom onset to admission, preexisting conditions, steroid, antibiotics, and vaccine (aHR = 5.27; 95% CI: 1.19, 14.50; P = 0.001) (**Table 4**).

**Table 4:** Associations of high-LDH with fatality of COVID-19 patients treated with Azvudine in Cox proportion hazard models.

Variable	Mode I^a^		Mode II^b^		Model III^c^		Model IV^d^	
	AHR [95%CI]	P	AHR [95%CI]	P	AHR [95%CI]	P	AHR [95%CI]	P
Composite disease progression outcome	4.96(1.89-13.06)	0.001	4.68(1.78-12.34)	0.002	4.98(1.88-13.16)	0.001	5.27(1.91-14.50)	0.001

LDH, lactate dehydrogenase.

a Unadjusted.

b Adjusted for age and sex.

c Adjusted for steroid and antibiotics.

d Adjusted for age, sex, severe, time from symptom onset to admission, preexisting conditions, steroid, antibiotics, vaccine.

## Discussion

The global outbreak of COVID-19 has caused significant fear and concern (Makević et al., 2022; Huang et al., 2023).. In response, scientists and researchers have developed vaccines to prevent infection and new drugs to treat infected individuals. Azvudine, as the first domestic oral antiviral agent approved in China, has been reported to shorten the time of nucleic acid negative conversion and cure patients with both common and severe COVID-19 (Ren et al., 2020; Yu and Chang, 2022; Shen et al., 2023).

In this retrospective cohort study, we found that COVID-19 patients treated with Azvudine had a relatively high proportion (33.9%) of high LDH levels, and demonstrated that high LDH levels was associated with alterations in laboratory markers. Moreover, individuals with high LDH levels upon SARS-CoV-2 infection require more intensive supportive treatment at the time of diagnosis and generally have a poorer prognosis compared to those with normal LDH levels.

Elevated LDH levels are indicative of tissue or cellular damage, making it a common marker for tissue injury. LDH elevation is also commonly observed during viral infections including MERS-CoV (Alsolamy, 2015; Al Ghamdi et al., 2016), H7N9 (Shi et al., 2013), and H5N1 (Oner et al., 2006). Similarly, in many severe cases of COVID-19, increased LDH activity has also been observed, serving as a marker of disease severity (Sidhwani et al., 2023). Previous studies have shown that after SARS-CoV-2 infection, there is an increase in white blood cell count, especially neutrophil count, leading to excessive cytokine production, cytokine storm, and systemic organ damage (Fialek et al., 2022). In our study, laboratory examination results upon hospital admission showed that COVID-19 patients in the high LDH group had higher neutrophil counts and increased proportions of white blood cells compared to the low LDH group. This may explain why patients with high LDH are more susceptible to pathogen infection due to weakened immune function following viral infection. We also found that patients with high LDH levels often exhibited decreased lymphocyte and eosinophil counts, indicating a possible correlation between lymphocyte damage and LDH release. Previous research has shown that inflammatory cytokines can increase LDH release from cells. For example, TNF-alpha can increase LDH release from Raji cells (Jurisic et al., 2004; Oner et al., 2006; Fialek et al., 2022; Sidhwani et al., 2023). The study conducted by Khalid et al. (2023) demonstrated a significant increase in the levels of IL-6, CRP, and PCT in patients with severe and critical conditions following SARS-CoV-2 infection. In our study, we further observed elevated serum concentrations of IL-6, IL-10, CRP, and PCT in patients with high LDH levels, suggesting a potential association between inflammatory cytokine release and LDH. Compared to the normal LDH group, patients with high LDH levels also demonstrated elevated levels of organ function markers such as DBIL, ALT, AST, SCr, BUN, CK, CK-MB, and BNP. In terms of coagulation function markers, patients in the high LDH group showed higher levels of D-dimer and FIB. Overall, our study results suggest that patients with high LDH levels may be at risk of a heightened inflammatory state and multi-organ dysfunction, which aligns with the Shi et al.’ conclusions (Shi et al., 2013).

Although there were no significant differences in disease severity between patients with or without high LDH levels, patients with high LDH levels tended to receive more immunoregulator and corticosteroid treatment and mechanical ventilation. The Cox regression model indicated that high LDH levels was an independent predictor for the composite disease progression outcome of COVID-19 patients treated with Azvudine, even after adjusting for potential confounders. These results suggest that high LDH levels may be a candidate biomarker for worse prognosis in patients treated with Azvudine.

To our best knowledge, this is the first study to report an association between high LDH levels and outcomes in patients with COVID-19 treated with Azvudine. However, several limitations deserve attention. Firstly, the LDH isoenzymes or LDH subunits were not tested due to limited resources. LDH isoenzyme or LDH subunits analysis in the future may help identify the source of increased LDH. Secondly, due to the massive number of patients and the lack of medical resources, the interval from illness onset to hospital admission was more than 5 days for most patients, which could have implications for disease progression and outcomes. Nevertheless, patients with high LDH levels had a similar interval from illness onset to hospital admission compared to those without normal LDH levels. Thirdly, although the data were consecutively collected and adjusted for a large number of confounders, we could not exclude the possibility of selection bias or confounding by indication due to the nature of retrospective cohort design.

In conclusion, we clarified the correlation between LDH levels and the prognosis of COVID-19 patients treated with Azvudine, and found that high LDH levels were associated with poorer short-term outcomes in COVID-19 patients treated with Azvudine. Therefore, stronger personal prophylactic strategies are advised for patients with high LDH levels, and more intensive surveillance and treatment should be considered when they are infected with SARS-CoV-2, especially for geriatric patients or those with preexisting comorbidities.

## Data availability statement

The original contributions presented in the study are included in the article/supplementary material. Further inquiries can be directed to the corresponding authors.

## Ethics statement

The studies involving humans were approved by Xiangya Hospital, Central South University (202002024). The studies were conducted in accordance with the local legislation and institutional requirements. The ethics committee/institutional review board waived the requirement of written informed consent for participation from the participants or the participants’ legal guardians/next of kin because This study was approved by the institutional review board of Xiangya Hospital, Central South University (202002024), and individual patient-informed consent was not required for this retrospective cohort study using anonymized data.

## Author contributions

Conception and design: GD, WZ, WC. Acquisition of data: MM, YS, YD. Interpretation of data, statistical analysis and manuscript writing: GD, WZ, WC. Revision of manuscript and administrative, technical, or material support: GD, WZ, WC.
